# Relationship between night-sleep duration and risk for depression among middle-aged and older people: A dose–response meta-analysis

**DOI:** 10.3389/fphys.2023.1085091

**Published:** 2023-03-02

**Authors:** Xin-lin Li, Jiayin Wei, Xinying Zhang, Zhuo Meng, Wentao Zhu

**Affiliations:** ^1^ School of Traditional Chinese Medicine, Beijing University of Chinese Medicine, Beijing, China; ^2^ School of Management, Beijing University of Chinese Medicine, Beijing, China

**Keywords:** night-sleep duration, sleep duration, depression, dose–response relationship, meta-analysis

## Abstract

**Objective:** The study aimed to examine the dose–response associations between night-sleep duration and depression risk in middle-aged and older adults.

**Methods:** We searched PubMed, Embase, Web of Science, CNKI, VIP, and the Wanfang data knowledge service platforms from inception to 31 July 2022. Cohort and case–control studies assessing the relationship between night-sleep duration and depression were selected. We used the Newcastle–Ottawa scale to assess the quality of the published research. Two researchers carried out data extraction and quality assessment. The restricted cubic spline function and generalized least squares method were used to establish dose–response relationships between sleep duration and depression. We aimed to analyze the estimated effect size presented as the risk ratio (RR) and its 95% confidence interval (CI) using Stata 12.0.

**Result:** Six cohort studies with 33,595 participants were included in this meta-analysis. A U-shaped association between sleep duration and depression risk was revealed. On one hand, compared with 7-h of night sleep, both shorter and longer sleep duration were associated with an increased risk of depression (5 h: risk ratio = 1.09, 95% confidence interval = 1.07–1.12; 6 h: RR = 1.03, 95% CI = 1.02–1.04; 8 h: RR = 1.10, 95% CI = 1.05–1.15; 9 h: RR = 1.31, 95% CI = 1.17–1.47; 10 h: RR = 1.59, 95% CI = 1.31–1.92; non-linear test *p <* 0.05). On the other hand, an increased risk of depression with shorter sleep duration was observed in middle-aged and older people among the non-Asian population (5 h: RR = 1.09; 95% CI = 1.02–1.17), while both shorter and longer sleep duration can increase the risk of depression among an Asian population (5 h: RR = 1.10, 95% CI = 1.07–1.13; 6 h: RR = 1.04, 95% CI = 1.02–1.05; 8 h: RR = 1.09, 95% CI = 1.05–1.14; 9 h: RR = 1.35, 95% CI = 1.18–1.53; 10 h: RR = 1.70, 95% CI = 1.36–2.12).

**Conclusion:** The lowest-risk onset of depression occurred among middle-aged and older people with 7 h of night sleep, which suggested that shorter and longer night-sleep duration might lead to an increased incidence of depression.

**Clinical Trial Registration:** https://www.crd.york.ac.uk/prospero/display_record.php?RecordID=344052, identifier 344052

## 1 Introduction

With the aging of the global population, mental health conditions such as depression have received greater emphasis among the middle-aged and older population. In 2008, the World Health Organization (WHO) pointed out that depression had become the third leading cause of the global disease burden and would develop into the largest by 2030 (Malhi and Mann, 2018). Meanwhile, depression is a recurrent mental disorder in middle-aged and older people, greatly impacting an individual’s psychosocial function. Depression is associated with numerous adverse health problems (e.g., disability, physical impairment, coronary heart disease, and increased mortality) (Carney and Freedland, 2017; Wu et al., 2023) and may threaten their lives and reduce their quality of life. In view of the depression-related high-level global health burden and economic burden (Bao et al., 2017; Paudel et al., 2013; van MILL et al., 2014), the occurrence of depression in middle-aged and older people has gradually captured more attention (Kang et al., 2020). Previous meta-analyses reported depression prevalence in middle-aged and older adults ranging from 8% to 16%, while the global prevalence of depression was 4.7% (4.4%–5.0%) (Cole and Dendukuri, 2003; Ferrari et al., 2013). Although several effective treatments for depression are available, more than 75% of patients from low- and middle-income countries do not receive any therapies (Evans-Lacko et al., 2018). However, only 30%–50% of the older patients accepting treatment can achieve remission (Lee et al., 2013). Hence, there is still a lack of health resources or timely diagnosis for middle-aged and older people who suffer from depression. In this situation, identifying modifiable risk factors for depression may provide implications for depression-related prognosis and primary prevention.

Many factors can account for depression, such as demographic and sociodemographic factors (e.g., age, gender, and lack of social support), and behavioral and psychosocial factors (e.g., low cognitive function, insufficient physical activity, and living alone) (Kang et al., 2020; Jiang and Chu, 2021). Insufficient sleep, short night-sleep duration, insomnia, and sleep disorders are highly relevant to high-risk depression (Paudel et al., 2013; van MILL et al., 2014; Fernandez-Mendoza et al., 2015; Gehrman et al., 2013). The relationship between sleep duration and depression is bidirectional and complex. On one hand, extreme sleep time is usually considered a symptom of depression. On the other hand, poor sleep is not only a symptom but also a risk factor for depression (Mendlewicz, 2009; Wiebe et al., 2012). Nocturnal sleep is a health-promoting physical process that plays a fundamental role in physical and mental fitness (Luyster et al., 2012). The incidence of sleep insufficiency is common in older people (Ancoli-Israel, 2005). Many cohort and cross-sectional studies showed a relationship between sleep duration and depression (Szklo-Coxe et al., 2010; Liang et al., 2020), and short sleep duration can increase the incidence of depression in adolescents (Gangwisch et al., 2010), adults (Watson et al., 2014), and older adults (Putilov, 2013). In addition, a U-shaped relationship between survival and actigraphically measured sleep duration, including short and long durations, was found (Kripke et al., 2011). In other words, long sleepers were also at risk for depression (Ford and Kamerow, 1989; Watson et al., 2015). Another Chinese study reported older people who slept more than 10 h per night had the highest risk of depression and major depressive disorder in rural areas (Jiang et al., 2020). Notably, the aforementioned research demonstrated a non-linear relationship between sleep duration and depression, but their conclusions about whether long or short sleep duration could induce depression were inconsistent. More importantly, few meta-analyses focused on cohort studies have been carried out to explore quantitative length of sleep duration and their impact on the risk of depression.

The study aims to analyze the relationship between night-sleep duration and the risk of depression through a dose–response meta-analysis based on cohort and case–control studies that quantitatively describe how night-sleep time influences the risk of depression in a bid to offer some clues to prevention for depression.

## 2 Materials and methods

### 2.1 Search strategy

First, we systematically searched eligible studies, including cohort and case–control studies, in PubMed, Embase, Web of Science, the China Network Knowledge Infrastructure (CNKI; 1979–2022), the Chinese VIP Information (1989–2022), and the Wan Fang database (1995–2022) from inception to 31 July 2022, limiting language to English and Chinese. Second, a series of topic-related editorials, perspectives, methodology, and comments were also reviewed for potentially useful information. Third, other relevant studies were manually investigated as the supplement literature. Our search terms encompassed “sleep duration,” “sleep time,” “sleep disorders,” “night sleep,” “sleepiness,” “sleep pattern,” and “depression.” Details of the systematic search are shown in Supplementary Table S1.

### 2.2 Selected criteria

The following original studies were considered for this meta-analysis: 1) middle-aged or older people as subjects; 2) epidemiological studies based on cohort population, case–control, and cross-sectional studies; 3) sleep duration with more than three exposure categories; 4) depression regarded as the endpoint for evaluation; 5) study providing odds ratio (OR), risk ratio (RR), or hazard ratio (HR) with 95% confidence intervals (CIs), or other sufficient data to estimate these values.

The exclusion criteria were as follows: 1) studies without full text available (conference proceedings) and review articles; 2) studies missing vital data for meta-analysis (e.g., number of cases, follow-up duration, RR/OR/HR value, and their 95% CIs) and inaccessible to contact with authors *via* email or telephone.

### 2.3 Data extraction

After two rounds of literature screening (title–abstract and full-text review) by two authors (Li XL and Zhang XY) independently in accord with the inclusion and exclusion criteria, irrelevant studies were removed. Any inconsistencies during this process were resolved by the third author (Zhu WT) through discussion. Additionally, we contacted authors for more detail *via* email address or telephone number if necessary. Subsequently, the core data were refined in a pre-set table (Table 1) covering the baseline characteristics of the population (e.g., age of participants or age range, and sample size.), stage of sleep duration (median of sleep group or the corresponding upper and lower boundaries), diagnostic criteria Newcastle–Ottawa Scale (NOS) scores, effect size (OR, RR, and HR), and the 95% CIs in each category of sleep duration.

**TABLE 1:** Characteristics of included studies.

Study ID	Country	Age range/mean age	No. of participants	Sleep duration levels, h	OR, (95% CI)	Diagnostic criteria	Adjustment	NOS score
Zhou (2021)	China	65.2 ± 7.1	16,104	<5	2.31 (1.81, 2.96)	Geriatric Depression Scale (GDS)	1–7, 9, and 16–21	7
				5–6	1.59 (1.36, 1.85)			
				7–8	1.00(Ref)			
				>8	0.67 (0.44, 1.04)			
Li (2018)	China	57.65 ± 8.67	6,948	<6	1.45 (1.30, 1.61)	Depression Scale (CES-D)	-	6
				6–7	1.07 (0.95, 1.21)			
				7–8	1(Ref)			
				>8	1.16 (0.99, 1.36)			
Lai et al. (2020)	China	65–101	2,620	≤5	0.84 (0.56, 1.27)	Hospital Anxiety Depression Scale (HADS)	1–6, 9, and 34–43	6
				6–7	1(Ref)			
				≥8	1.79 (1.24, 2.59)			
Lippman et al. (2017)	America	>50	1,110	<6	1.8 (1.3, 2.6)	Depression Scale (CES-D)	1–6, 8-12, 22, and 25–28	7
				6–8	1(Ref)			
				≥9	1.1 (0.7, 1.9)			
Reis et al. (2018)	Portugal	≥65	1816	≤5	1.03 (0.64, 1.66)	Not report	2, 4, and 15	6
				6–8	1(Ref)			
				≥9	1.34 (0.68, 2.64)			
Yokoyama et al. (2010)	Japan	≥65	4,997	<6	0.875 (0.508, 1.505)	Depression Scale (CES-D)	29–33	8
				6–7	1.259 (0.851, 1.865)			
				7–8	1(Ref)			
				8–9	1.218 (0.842, 1.764)			
				≥9	1.126 (0.693, 1.828)			

Adjustments: 1, age; 2, sex; 3, body mass index (BMI); 4, educational status; 5, smoking; 6, alcohol; 7, occupation; 8, cardiac disease; 9, physical activity; 10, systolic blood pressure; 11, race–ethnicity; 12, diabetes; 13, diastolic blood pressure; 14, antihypertensive mediations; 15, NUTS II regions; 16, communication frequency with relatives; 17, quantity of chronic disease; 18, time of afternoon nap; 19, support from relatives; 20, income ratio; 21, self-perceived health status; 22, insurance; 23, time from sleep reports to CES-D; 24, time between CES-D 1 and CES-D, 2; 25, social isolation; 26, systolic blood pressure; 27, diastolic blood pressure; 28, antihypertensive medications; 29, excessive daytime sleepiness; 30, discomfort feeling in the legs; 31, difficulty initiating sleep; 32, early morning awakening; 33, difficulty maintaining sleep; 34, medical history; 35, Groningen Activity Restriction Scale (<19); 36, habitual snoring (no); 37, Athens Insomnia Scale (<5); 38, Epworth Sleepiness Scale (<10); 39, taking hypnotics in the past 1 month (no); 40, sleep duration (6 to 7 h); 41, bedtime (21:31–22:30); 42, rise time (05:00–06:00); 43, anxiety subscale (<3).

### 2.4 Quality assessment

Two authors (Wei JY and Meng Z) independently applied NOS to evaluate the quality of the included studies using different evaluation criteria for cohort and case–control studies. The NOS scale consists of three dimensions and eight items: four items for the selection of study subjects, one item for comparability between/among groups, and the remainder (three items) for measuring endpoint or exposure factors. Except for one item with possible two points for comparability, the others can have either one or no points as the highest score. Cohort studies had a maximum score of 9. Studies with a total score equal to or more than seven are considered high certainty, while studies with scores between four and six were defined as fair. Studies with a total score of less than four were considered low certainty (Stang, 2010). The third investigator (Zhu WT) made decisions when disagreements or questions arose.

### 2.5 Data analysis

First, the RR and its 95% CI were employed to estimate effect size, during which log (RR) was calculated by the RR values provided. If different effect sizes appeared in the included studies, OR and HR were directly treated as RR (Orsini et al., 2012). When studies reported multiple adjusted RR values, we extracted the most adjusted estimates. In line with the theory proposed by Greenland and Longnecker (1992), an MS Excel macro document (Hamling et al., 2008) was used to convert the lowest dose group set as a reference substance. When the lowest and the highest dose group were in an open interval, the length of the interval was defined as that of the adjacent groups, and the middle point of the interval was taken as the average exposure dose (Orsini et al., 2006). If the mean or median of the sleep duration group was not reported in the original text, the median of the upper and lower boundaries of the group was used instead. When including studies that had other missing data, we estimated those data with reference to previous studies (Bekkering et al., 2008).

Second, data from the highest and lowest dose groups in the included studies were extracted for high–low meta-analysis. When *p* < 0.05, there was a statistical difference in the risk of depression between the highest and lowest dose groups regarding night-sleep duration. In view of the aforesaid statistical differences, meta-regression was performed using the three-node restricted cubic spline (RCS) to fit the potential non-linear trend. The three knots were at 10th, 50th and 90th percentiles of the distribution. The generalized least-square method was used to estimate the parameters. Afterward, the common comparative category for the exposure was set to 7 h (von Ruesten et al., 2012). Compared with the reference dose, the RR and the 95% CI that described the relationship between different exposure doses and depression were estimated.

Third, the independent variable, night-sleep duration, was treated as a continuous variable in our study, and the log (OR) of depression was the dependent variable. The Wald test was used to determine non-linearity. The relationship between the two variables was considered to be a non-linear dose–response if *p <* 0.05. If a U, J, or S-shape of the non-linear model was observed, two piecewise treatments were respectively performed with the lowest point of the curve to show a trend identified as linearity (Xu et al., 2019; Liu et al., 2017).

Statistical heterogeneity was measured by Cochran’s Q-test employing the *I* ^2^ value (Higgins and Thompson, 2002). Q statistics with *p* < 0.1 presented heterogeneity. Meanwhile, if heterogeneity was indicated by *I* ^*2*^ ≥ 50% (Kang and Xu, 2015), then the random-effects model was applied. In contrast, the fixed-effect model approach was applied to estimate the coefficient. We executed a subgroup analysis considering different confounding factors such as gender and study location. Egger’s and Begg’s tests were employed to assess whether publication bias existed when more than 10 studies were included. Otherwise, neither Begg’s nor Egger’s test was carried out. For studies reporting unadjusted effect sizes or those of fair quality, sensitivity analysis (leave-one-out method) was utilized to quantify the distinction between before and after removing a single study. For all the tests except the Q-test, *p*-values of 0.05 were considered significant, using STATA version 12.0 (STATA Corporation, College Station, Texas, United States of America).

### 2.6 Registration and reporting

We conducted the meta-analysis according to the Meta-analysis of Observational Studies in Epidemiology (MOOSE) checklist (Stroup et al., 2000). The meta-analysis was registered on the PROSPERO platform (CRD42022344052).

## 3 Results

### 3.1 Description of studies

**FIGURE 1:**
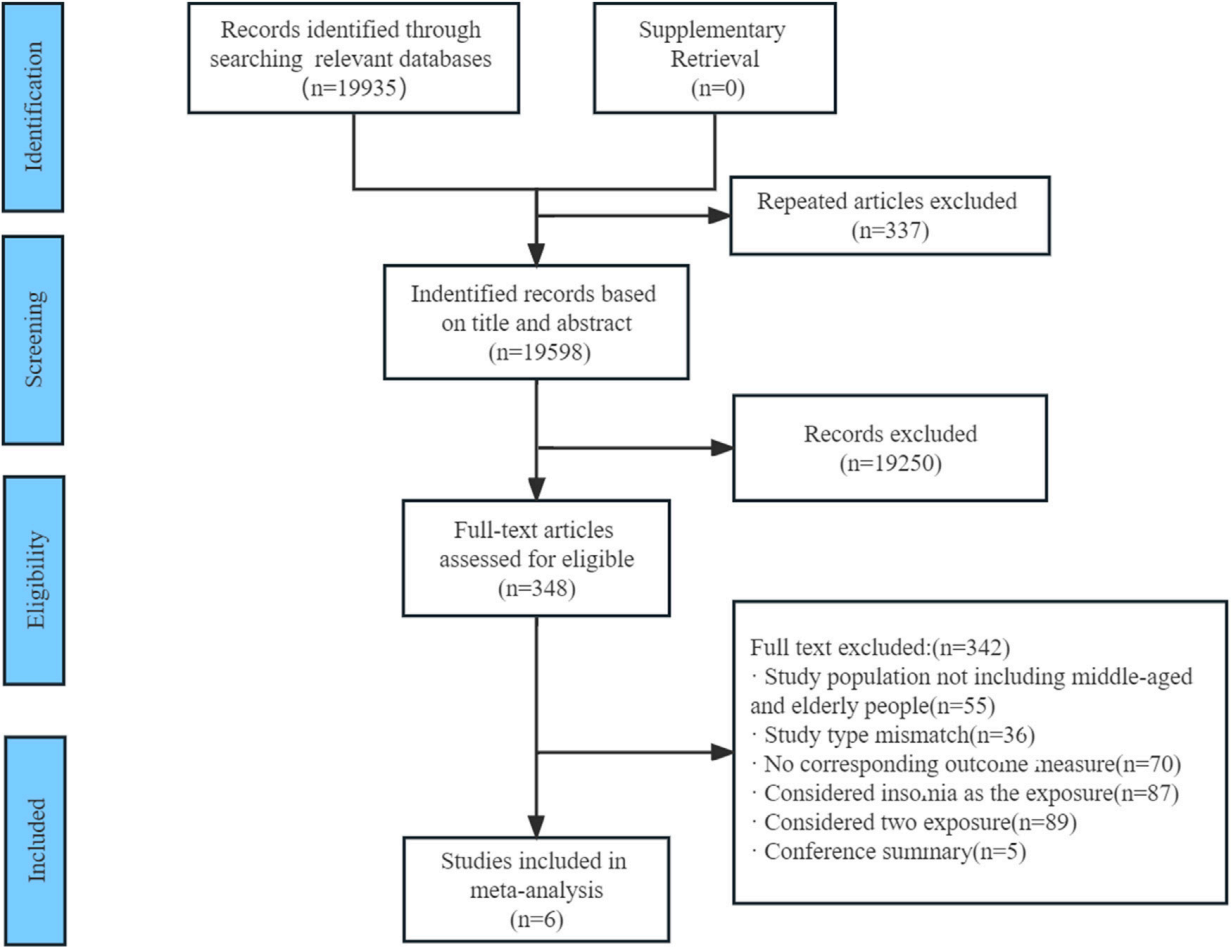
Flow diagram of the search strategy and study selection.

After preliminary searches in the databases mentioned previously, 19,935 studies were identified. After the first screening of the ineligible articles, we retrieved full-text papers of 348 studies for further identification. Of these, 341 studies were excluded according to the inclusion criteria. Finally, we included six cohort studies through cross-sectional investigation enrolling 33,595 participants (Yokoyama et al., 2010; Lippman et al., 2017; Li, 2018; Reis et al., 2018; Lai et al., 2020; Zhou, 2021). Figure 1 shows the selection process. These studies were published between 2010 and 2021. The details of their characteristics are shown in Table 1. The follow-up durations were 3–9 years. The sample size of cohort studies ranged from 1,110 to 16,104. Three of these cohort studies were carried out in China (Li, 2018; Lai et al., 2020; Zhou, 2021), and one each in America (Lippman et al., 2017), Portugal (Reis et al., 2018), and Japan (Yokoyama et al., 2010). These studies recruited men and women. Only one study reported the results of men and women separately (Li, 2018). In terms of the depression measurement, three studies considered the Center for Epidemiological Survey, Depression Scale (CES-D) as the diagnosed standard (Yokoyama et al., 2010; Lippman et al., 2017; Li, 2018), one study diagnosed depression based on the Geriatric Depression Scale (Zhou, 2021), and one study (Lai et al., 2020) used the Hospital Anxiety and Depression Scale (HADS) as diagnostic instruments. Only one study did not report the diagnostic criteria for depression (Reis et al., 2018). The risk of bias (accessed by NOS proposed previously) of the included studies was generally good; three studies were considered fair because of failure to ascertain exposure, short length of follow-up, and outcome of interest not present at the start of the study. Details of the quality assessment are shown in Supplementary Table S2.

### 3.2 The impact of night-sleep duration and depression in middle-aged and older people

**FIGURE 2:**
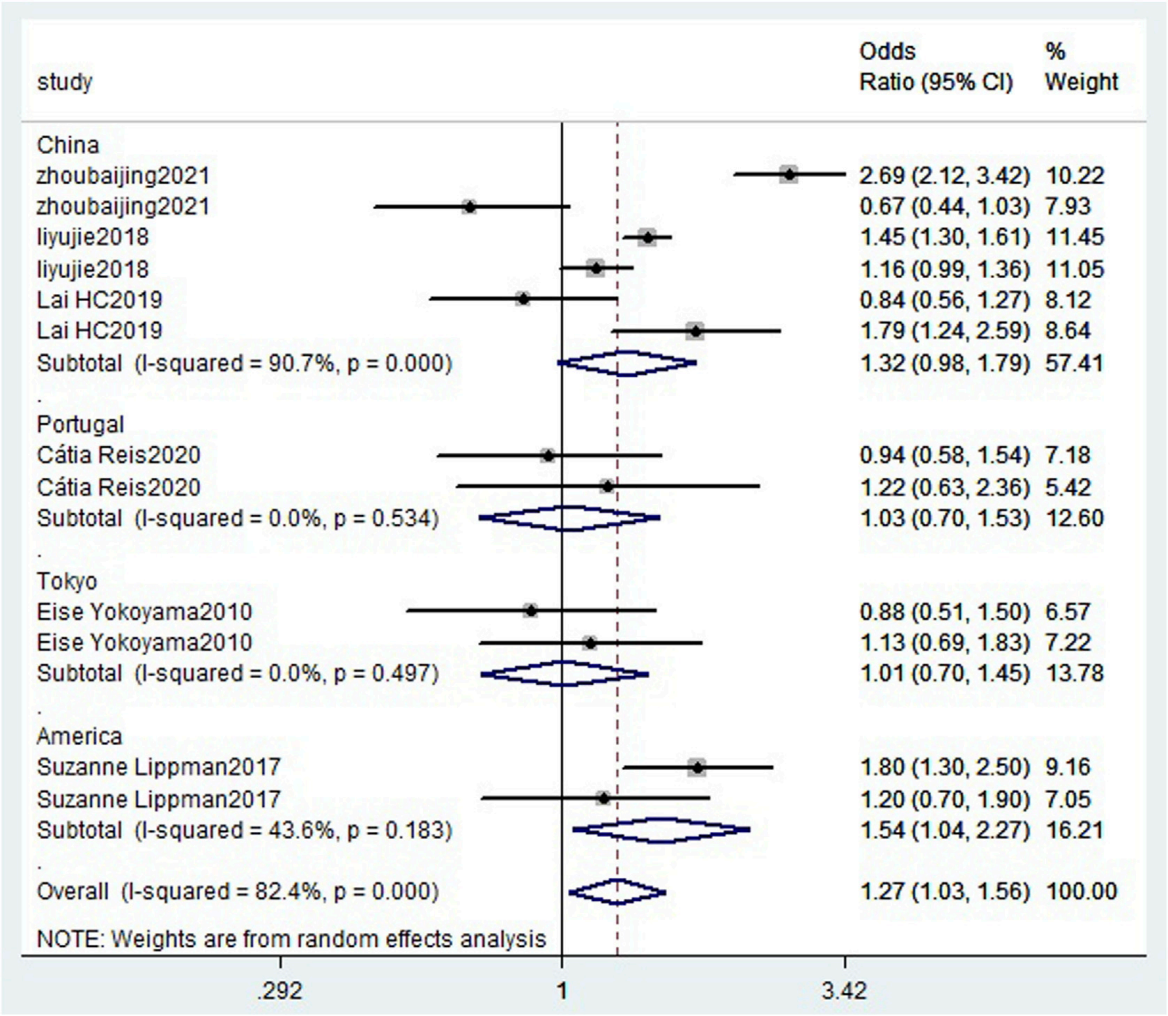
High-low meta-analysis forest chart between sleep duration and depression risk.

Data reported in six eligible studies (Yokoyama et al., 2010; Lippman et al., 2017; Li, 2018; Reis et al., 2018; Lai et al., 2020; Zhou, 2021) were utilized for a high- and low-dose meta-analysis. The fixed-effect model was used for consolidation because of heterogeneity (*I* ^2^ = 0.0% and *p* = 0.884). The results showed that there was a statistically significant difference in the risk of depression between long sleep and short sleep (RR = 1.27, 95% CI = 1.03–1.56; *p* = 0.000; Figure 2).

**FIGURE 3:**
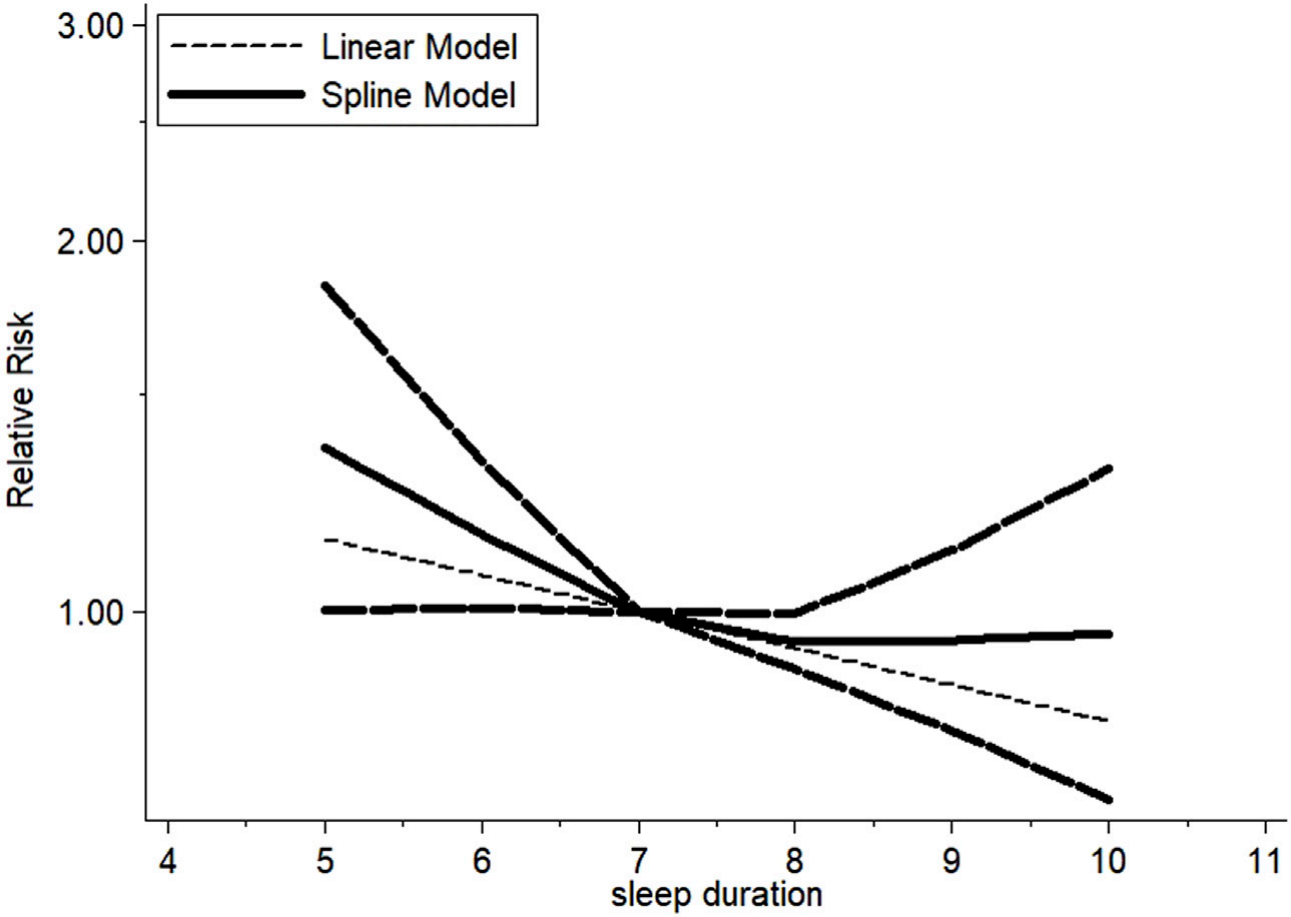
Dose–response meta-analysis of sleep duration and depression risk.

Because there were statistical differences in the risk of depression in the long and short sleep time groups, a dose–response meta-analysis was conducted for the six cohort studies. There was a non-linear relationship estimating a U-shaped curve between sleep duration and the risk of depression in middle-aged and older people (non-linear test *p* < 0.05; Figure 3). When the sleep duration was 7 h, the risk of depression was the lowest. Compared to 7 h, shorter and longer sleep durations were significantly associated with the risk of depression during night-sleep time (5 h: RR = 1.09, 95% CI = 1.07–1.12; 6 h: RR = 1.03, 95% CI = 1.02–1.04; 8 h: RR = 1.10, 95% CI = 1.05–1.15; 9 h: RR = 1.31, 95% CI = 1.17–1.47; 10 h: RR = 1.59, 95% CI = 1.31–1.92).

### 3.3 Subgroup analyses of night-sleep duration and depression

The association between the duration of night sleep and the incidence of depression may be inconsistent in middle-aged and older people of different genders and from different regions, so a subgroup analysis was required. Effect sizes were used to estimate the effect of gender and regional factors on the dose–response relationship between sleep duration and depression incidence.

We examined the effect through applied subgroup analysis based on the gender and region. Compared to 7 h, a shorter duration of 5 h led to an increased risk of depression in a non-Asian older population (RR = 1.09; 95% CI = 1.02–1.17). Among older Asian adults, sleeping less than and more than 7 h was associated with an increased risk of depression (5 h: RR = 1.10, 95% CI = 1.07–1.13; 6 h: RR = 1.04, 95% CI = 1.02–1.05; 8 h: RR = 1.09, 95% CI = 1.05–1.14; 9 h: RR = 1.35; 95% CI = 1.18–1.53; 10 h: RR = 1.70, 95%CI = 1.36–2.12). The results are shown in Table 2.

**TABLE 2:** Dose–response meta-subgroup analysis of sleep duration and risk of depression.

Subject	Number of included studies	Night-sleep duration					
		5	6	7	8	9	10
		RR (95% CI)	RR (95% CI)	RR (95% CI)	RR (95% CI)	RR (95% CI)	RR (95% CI)
Region							
Asia	4	1.10 (1.07,1.13)	1.04 (1.02,1.05)	1.00 (1.00,1.00)	1.09 (1.05,1.14)	1.35 (1.18,1.53)	1.70 (1.36,2.12)
Non-Asia	2	1.09 (1.02,1.17)	-	1.00 (1.00,1.00)	-	-	1.16 (0.73,1.85)

Note: Evaluated by non-linear trends.

Only Li,(2018) reported the relationship separately for men and women. This study showed that a duration of 6 h would increase the risk for both groups (RR = 1.40, 95% CI = 1.09–1.79), while in women, sleeping <7 h was also a risk factor for depression (RR = 1.378, 95% CI = 1.11, 1.72).

### 3.4 Sensitivity analyses

Sensitivity analysis was performed to test the stability of the meta-analysis result. After excluding fair-quality studies (n = 3), there was no particular study with a significant impact on the overall estimates under sensitivity analysis (Supplementary Figure S1).

### 3.5 Publication bias

Due to the small number of included studies (<10), we did not conduct a visual inspection of Begg’s funnel plot or statistical Begg’s and Egger’s tests for detecting publication bias.

## 4 Discussion

### 4.1 Summary of findings

Evidence of a dose–response association between night-sleep duration and depression has been inconsistent and limited. We conducted this study to investigate the non-linear impact of sleep duration on depression in middle-aged and older people. We performed a dose–response meta-analysis based on cohort studies with an average sleep duration of 5 to 10 h. Our results suggested that the incidence of depression was related to sleep duration, with a dose–response relationship between night-sleep duration and the risk of depression. Compared with 7 h, both shorter and longer sleep duration could influence the risk of depression. The significant association remained the same in subgroup analyses. Further subgroup analysis of regions showed that short sleep duration increased the risk of depression, regardless of race (Asian and non-Asian populations), while sleepers with a longer duration from Asia also gained a significantly increased risk of depression. Therefore, regulating sleep duration is important for the prevention and intervention of depression.

### 4.2 Comparison with existing meta-analyses

Data from Australia, the United States, and Germany showed that more than 30% of adults had trouble sleeping (Jike et al., 2018). Sleep disturbances that include insomnia, poor sleep qualities, and sleep complaints (Yaffe et al., 2014) were classified according to symptoms, questionnaires, and diagnoses. A previous review of 34 cohort studies investigating the relationship between insomnia and depression showed that sleep difficulty was significantly associated with depression (Li et al., 2016), which suggested that individuals experiencing insomnia may be at higher risk of depression than people who sleep for a longer duration. Furthermore, some meta-analyses emphasized depression and poor sleep quality as an unfavorable effect on older individuals (Becker et al., 2017). Bao et al. (2017), including 23 cohort studies, reported that sleep disturbances were associated with an increased risk of depression. Zhai et al. (2015) indicated that both shorter and longer sleep duration, without reporting baseline sleep time, were significantly associated with an increased risk of depression in adults. However, these studies did not report the impact of specific sleep duration on the risk of depression. Thus, we conducted this meta-analysis using a flexible, non-linear, meta-regression approach, which showed our results were consistent with previous meta-analyses: both long and short night-sleep duration contribute to a risk of depression. Concretely, toward gender analysis, shorter sleep duration contributes to a higher risk of depression (Li, 2018).

### 4.3 Relationship between sleep duration and depression

Depression is related to decreased regional gray matter volume, which might partly be explained by an unhealthier lifestyle in depressed individuals (Binnewies et al., 2021). Undoubtedly, the length of sleep is vital for inflammation, glucose regulation, energy expenditure, and cognitive development. One possible explanation of sleep duration regarded as an intermediary variable of depression is that continuous changes in the arousal system accompanied by stress will cause sleep problems. When these changes become normal, the emotional regulation or cognitive system functions will gradually change, resulting in depression symptoms (Magnusson et al., 2014). Short sleep may increase daytime physical or psychological fatigue (Shen et al., 2006), promoting the onset of depression, which leads to biological cycle rhythm disorder or causes endocrine hormone changes (Germain and Kupfer, 2008; van Noorden et al., 2012; Luik et al., 2015). The bi-directional relationship between insomnia and depression has previously been established (Bao et al., 2017; Nutt et al., 2008). Furthermore, people who sleep for a long time still have a risk of depression, which may be correlated with low physical activity (Stranges et al., 2008). Decreased activity will reduce the level of neurotransmitters (especially dopamine and serotonin), the transmission of aminergic brain synapses (Weicker and Struder, 2001), and endorphin secretion (Janal et al., 1984), which will increase the risk of depression (Paluska and Schwenk, 2000).

### 4.4 Relationship between gender and depression

Previous studies found that women are more likely to be affected by sleep duration, implying that gender could be another risk factor for depression (Wang et al., 2016). Women are more susceptible to the synergistic effect of hormones than men (such as estrogen) during most stages (*viz.* puberty, premenstrual period, *postpartum* period, and the transitional period of perimenopause), so they are prone to be exposed to depression (Janowsky and Rausch, 1985). Because only one study reported results for men and women separately, it is not enough to draw an accurate conclusion about the relationship between different genders and depression. Further research investigating the impact of sleep duration and depression on gender should be encouraged.

### 4.5 Strengths and limitations

There were a few strengths in this work. First, our review, with a strict inclusion of eligible cohort studies, applied the RCS function to fitting the potential non-linear relationship between sleep duration and the risk of depression. To some extent, the results present a cause and effect relationship. In the second place, this manuscript revealed the relationship between specific sleep duration and the risk of depression, which complemented stronger and more sufficient evidence. Lastly, sensitivity analysis was carried out to identify the stability of our results, probably affected by underlying confounders, while subgroup analysis was run to detect sources of heterogeneity. As a result, repeated consistency after sensitivity and subgroup analyses indicated that our findings are relatively reliable and robust.

However, we must explain our findings carefully, owing to several limitations. First, this review only includes studies written in Chinese and English and may have language bias. Second, only six cohort studies were entered into the meta-analysis finally, which could affect the power of the results. Due to the limited number of studies included, it was not possible to analyze subgroups according to the number of participants, follow-up years, case sources, *etc.* Although we adopted subgroup analyses of Asian and non-Asian regions, the small number of included studies in the two regions limited the generalization to a certain extent. Third, this meta-analysis contained cohort studies addressing the relationship between night-sleep duration with the risk of depression, regardless of daytime sleepiness. Sleep duration collected through questionnaires in some studies may be biased, owing to the inaccurate records of older people. The adjusted confounders were different among the included studies. Although we extracted the adjusted risk estimates, it was difficult to avoid information bias derived from original studies. Due to the limited number of related studies, publication bias was not examined. An updated meta-analysis adopting more studies should be undertaken in the future.

## 5 Conclusion

The lowest incidence rate of depression occurs in middle-aged and older people who sleep 7 h a night. Compared with 7-h sleep duration, shorter and longer night-sleep duration could lead to an increased risk for depression. Hence, it is necessary to avoid both oversleeping and under-sleeping to protect individuals against depression.

## Data Availability

The original contributions presented in the study are included in the article/Supplementary Material; further inquiries can be directed to the corresponding author.
