# Supplementary material for: Relationship between night-sleep duration and risk for depression among middle-aged and older people: A dose–response meta-analysis
Source: Front Physiol. 2023 Mar 2;14:1085091. doi: 10.3389/fphys.2023.1085091 (PMC10017495; doi:10.3389/fphys.2023.1085091)
Supplement: Supplementary file 1 [file Table1.docx]

***Supplementary material***

***Sleep duration in relation to depression in elder adults: A dose-response meta-analysis***

***Xinlin Li et al.***

***Supplemental Table 1. Terms were used in the search***

| ***Database*** | ***Syntax*** | ***Results*** |
| --- | --- | --- |
| *PubMed* | *("sleep duration "[Title/Abstract] OR "sleep time"[Title/Abstract] OR sleep disorders [Title/Abstract] OR night sleep[Title/Abstract] OR sleepiness[Title/Abstract] OR sleep pattern[Title/Abstract]) AND ("depression*"[Title/Abstract])* | *86* |
| *Web Of Sciences* | *TI=("sleep duration" OR "sleep time" OR "sleep disorders" OR "night sleep" OR "sleepiness" OR sleep pattern) AND TS=( " depression ")* | *2520* |
| *Embase* | *(sleep duration:ti,ab,kw OR sleep time:ti,ab,kw OR sleep disorders:ti,ab,kw OR night sleep:ti,ab,kw OR sleepiness:ti,ab,kw) AND ('depression:ti,ab,kw)* | *16645* |
| *CNKI* | *SU%=sleep time and SU%=depression* | *337* |
| *Wanfang* | *Title or Keywords:(sleep time) and Title or Keywords:(depression)* | *339* |
| *VIP* | *M=sleep time and M= depression* |  |

***Supplemental Table 2 Detail of quality assessment of included studies based on Newcastle-Ottawa Scale***

| *Study ID* | *Selection* | | | | *Comparability* | *Outcome* | | | *Total score* |
| --- | --- | --- | --- | --- | --- | --- | --- | --- | --- |
|  | *Representativeness of the sample* | *Ascertainment of exposure* | *Selection of*  *non-exposed cohort* | *Outcome of interest not present at start of study* | *Control for important factor or additional factor* | *Assessment of outcome* | *Enough time to follow up* | *Adequacy of follow up* |  |
| *Zhou B2021* | * | * | * |  | * | * | * | * | 7 |
| *Li Y2018* | * |  | * |  | * | * | * | * | 6 |
| *Lai HC2019* | * | * | * |  | * | * |  | * | 6 |
| *Suzanne L2017* | * |  | * | * | ** | * | * |  | 7 |
| *Reis C 2018* | * |  | * | * | * | * |  | * | 6 |
| *Yokoyama E* *2010* | * | * | * | * | ** | * |  | * | 8 |
