# Supplementary figures and images for: Relationship between night-sleep duration and risk for depression among middle-aged and older people: A dose–response meta-analysis
Source: Front Physiol. 2023 Mar 2;14:1085091. doi: 10.3389/fphys.2023.1085091 (PMC10017495; doi:10.3389/fphys.2023.1085091)

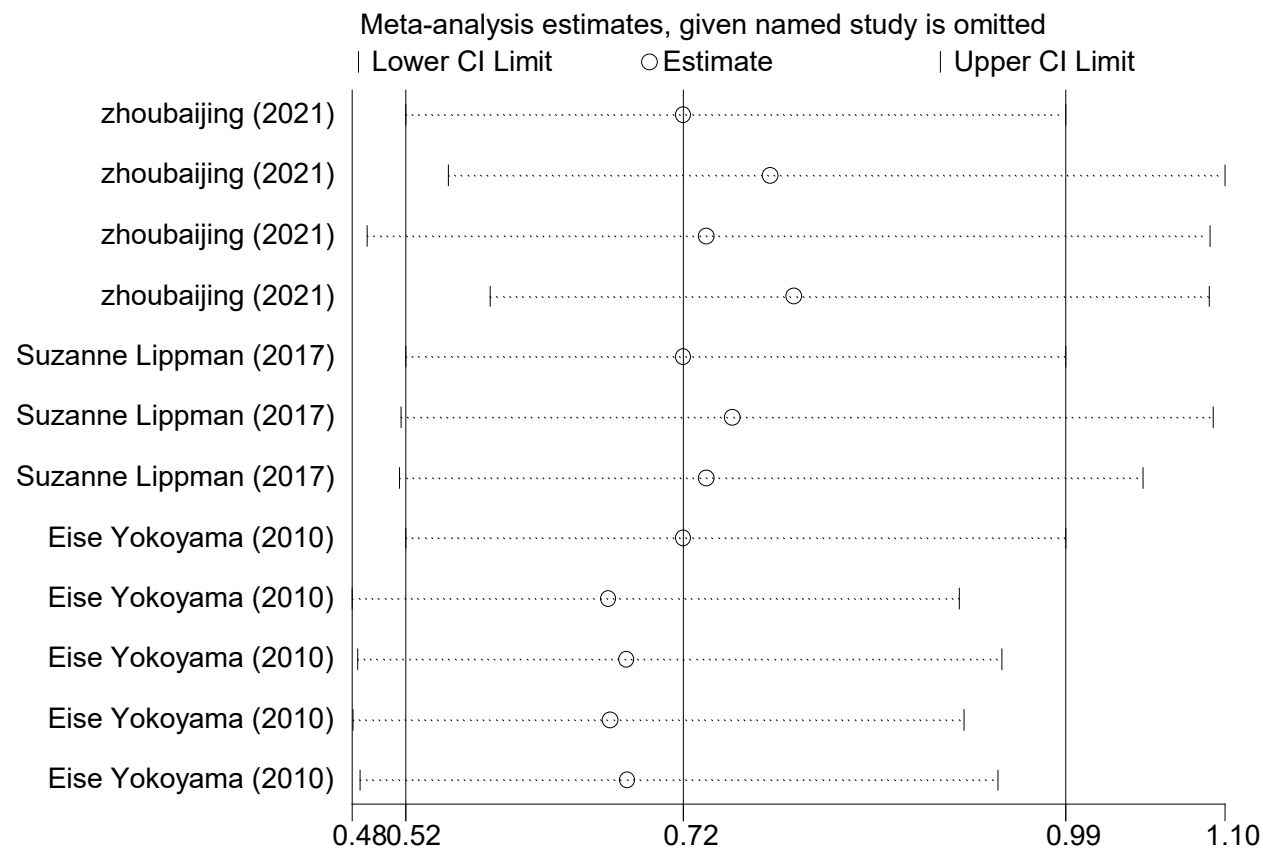

***Supplemental Figure 1 Results of included studies based on sensitivity analysis***

Supplement: Supplementary file 2 [file Image1.pdf]
